# Modularity in Biological Networks

**DOI:** 10.3389/fgene.2021.701331

**Published:** 2021-09-14

**Authors:** Sergio Antonio Alcalá-Corona, Santiago Sandoval-Motta, Jesús Espinal-Enríquez, Enrique Hernández-Lemus

**Affiliations:** ^1^Computational Genomics Division, National Institute of Genomic Medicine, Mexico City, Mexico; ^2^Centro de Ciencias de la Complejidad, Universidad Nacional Autónoma de México, Mexico City, Mexico; ^3^National Council on Science and Technology, Mexico City, Mexico

**Keywords:** modularity, community structure, motifs, biological networks, systems biology

## Abstract

Network modeling, from the ecological to the molecular scale has become an essential tool for studying the structure, dynamics and complex behavior of living systems. Graph representations of the relationships between biological components open up a wide variety of methods for discovering the mechanistic and functional properties of biological systems. Many biological networks are organized into a modular structure, so methods to discover such modules are essential if we are to understand the biological system as a whole. However, most of the methods used in biology to this end, have a limited applicability, as they are very specific to the system they were developed for. Conversely, from the statistical physics and network science perspective, graph modularity has been theoretically studied and several methods of a very general nature have been developed. It is our perspective that in particular for the modularity detection problem, biology and theoretical physics/network science are less connected than they should. The central goal of this review is to provide the necessary background and present the most applicable and pertinent methods for community detection in a way that motivates their further usage in biological research.

## 1. Introduction

The field of Systems Biology has many branches that focus on studying networks. It is common to encounter in the literature terms such as metabolic networks, transcriptional networks, protein-protein interaction networks, etc. These networks are graph-theoretical constructs composed of nodes and edges that aim to describe the integrated state of a biological system. Nodes represent the elements of the system, while edges represent the relation between any two of these elements. Depending on the scale of the biological entities at hand, a network can describe systems such as: ecological systems where each node is a biological entity itself; an organism with nodes being organs or groups of organs; tissues or individual cells with genes, proteins, organelles, and metabolites interacting with each other; down even to the level of amino acids interacting to build a protein. Networks facilitate the identification of relevant entities and interactions through the use of theoretical and computational analysis over experimental data. These analyses aim to make predictions, or at least detailed and accurate descriptions of the underlying biological systems. Since one of the most common applications of complex systems in biology is the representation of biological interactions as edges or links of a network, the connectivity or interaction structure of such a network is of utmost importance. This structure is known as the topology of the network and in biological systems it is usually not random. This means that who is connected to whom is relevant, and the distribution of links is arguably related to the particular functionality of such systems.

In biological systems, modularity has been associated with properties such as robustness (Aldana and Cluzel, 2003), mainly derived from the Boolean network approach (Kauffman, 1969). The concept of robustness is related to the ability of a system to withstand perturbations and retain its functionality, whichever it may be (Aldana et al., 2007). Examples of robustness in a biological system can be observed in biochemical networks (Barkai and Leibler, 1997; Morohashi et al., 2002), signaling networks (Igoshin et al., 2007; Espinal et al., 2011; Espinal-Enríquez et al., 2017) and other complex biosystems. For instance, in prokaryotic organisms, sigma factors, despite their structural similarity, regulate different sets of genes, but the regulatory function of a dysfunctional sigma factor can be reassigned to other sigma factors making the organism functional (Torres-Sosa et al., 2012). Another example of modularity arises when a set of genes is regulated by the same transcriptional factor (set known as a *regulon*). It has been proposed that these sets of genes can give rise to functional modules in *Pseudomonas aeruginosa* (Schulz et al., 2015) and that such modules are essential for the adaptation and survival under challenging environments.

One goal of studying biological systems as networks is to understand how the interconnectedness and function of each element derives in a system-level behavior. In order to uncover these features one can look into the *design principles* of the network. This means, to try to uncover the particular patterns present in the network's topology, such as the ways the nodes are connected to each other; the functional groups they belong to; or if nodes with a particular function agglomerate in subgroups. Topological features, of course, are only partially responsible for the actual design principles of biological systems. Connectivity features common of biological networks, such as the approximate scale-free nature of their connectivity distributions, hierarchical and modular organization, set the stage for functional features to emerge. Such functional features are a consequence of the underlying organizational structure of the systems, their physiological setting and environmental constraints. Regarding network connectivity, it is known that the organization patterns of large complex networks are often composed of structural sub-units often called modules or communities (Girvan and Newman, 2002). Communities and modules in the present context are interchangeable terms, however in this manuscript we will use the latter term as we believe it has a similar meaning over a large number of disciplines, with the possible exception of the Social Sciences and Mathematics.

## 2. Modularity in Biological Systems

So what is a module? Despite there is still no consensus on what defines a module, a generally accepted notion is that it corresponds to a tightly interconnected set of edges in a network. Intuitively, the density of connections inside any so-called module (*within-connections*) must be significantly higher than the density of connections with other modules (*between-connections*) (Thieffry and Romero, 1999; Girvan and Newman, 2002; Clauset et al., 2004; Palla et al., 2005). Modularity has been helpful in many biological fields and can even be useful in exploratory research (Serban, 2020). In the following sections, we will present and discuss the latest developments of modularity research in biological systems as well as the necessary concepts and formal definitions to understand and promote the usage of several modularity detection algorithms in the biological sciences (Didier et al., 2018; Li et al., 2019).

### 2.1. Emergence of Modularity

In order to perform their vital functions and at the same time comply with changing environmental conditions, living systems must possess a high degree of internal organization. A likely scheme to attain such a sophisticated degree of organization is through the coupling of diverse biological processes, which creates the needed correlations among their internal and external constraints to perform a certain task. This theory is known as the *networks of processes* (Clarke and Mittenthal, 1992) and suggests that modules can be thought as clusters of coupled elements that work under certain constraints. It also states that organisms can be studied as super-modules (e.g., networks) made up of several interplaying modules that adapt as a whole to changes in their environment. Under this scheme, modularity can be thought of as a very effective way to prioritize and optimize the correct functioning of living systems, which are undoubtedly subject to changing environmental conditions or even to entropic decay.

The question of how modularity emerges in biological networks has no definitive answer yet, either. It has been shown that dynamical networks, which include temporal processes occurring in the whole spatial structure of the network, can give rise to modular behavior when driven by growth, duplication and diversification. These duplication-centered dynamic models emerge from the fact that if some parts of a system undergo duplication, the new system will be more modular than the original (Lorenz et al., 2011). How modularity emerges is closely related to the question of how and why it is preserved across so many biological systems (Kashtan and Alon, 2005; Gibson, 2016). This question has been addressed in evolutionary/developmental biology (evo/devo) and in molecular systems biology as a kind of intersection point between both disciplines. It has been argued that there is indeed a relationship between modularity and controllability (Constantino and Daoutidis, 2019).

Despite underlying mutational mechanisms have been proposed to explain the emergence of modularity, selection and other evolutionary forces have also been part of this discussion (Wagner et al., 2001, 2007; Espinosa-Soto and Wagner, 2010; Clune et al., 2013; Friedlander et al., 2013; Banerjee et al., 2017; Verd et al., 2019; Jaeger and Monk, 2021), as are ecological factors such as spatial distribution and population dynamics (Gilarranz, 2020). Biological modularity arise in the contexts of dynamical process that may even challenge compartmentalization and cause the breakdown of modularity or its rearrangement (Valverde, 2017; Wang et al., 2021).

In the next section, we will discuss the different notions of modularity –particularly those more closely related to the modular organization at the molecular, functional and cellular levels– and their application to a wide diversity of biological phenomena.

### 2.2. Applications of Network Modularity

One clear example of application of network theory in biology is the study of Gene Regulatory Networks (GRNs) (Davidson and Levin, 2005). These networks can be conceptualized as control systems that drive whole-genome expression patterns (Hernández-Lemus et al., 2019). This coordinated expression is attained through the orchestrated expression of transcription factors and other regulatory molecules like siRNAs, histones, etc. The wider availability of high throughput technologies has sprouted a new wave of modularity research in GRNs. After the completion of the human genome project (HGP), and following the pioneering work of Kauffman (1969) and Britten and Davidson (1969) in the late 1960s, transcriptional regulation module discovery has become an extremely fruitful research field. For instance, it has been demonstrated that modularity can emerge as a consequence of gene co-expression in GRNs; by associating the functions of these genes and their regulators, it has been argued that gene co-expression may confer functional advantages to the organisms, as genes with related functions are likely regulated in a similar manner (Solé et al., 2002; Narula et al., 2010). Gene functionality of several genes with no prior functional description has already been predicted (Segal et al., 2003; Lee et al., 2004; Tanay et al., 2004). Also, by integrating gene expression levels with the modular structure, it was possible to build a comprehensive map of gene regulation for a whole organism (Zhu et al., 2008).

Community structure and modularity in metabolic networks is another important research field. Many biochemical interventions and biotechnological applications depend on modularity, and with the advent of synthetic biology, the use of modules will probably escalate in the near future, driven by the possibility to evolve engineered biological systems (Parter et al., 2007). Modularity in metabolic networks has been extensively explored since the pioneering work by Ravasz et al. (2002) where through the reconstruction of 43 metabolic networks from different organisms, they found that scale free topologies were ubiquitous. Briefly, in these networks the probability distribution of connections on the network (degree-distribution) follows a power law, so that most nodes will end up with few connections and only a few nodes will end up with many. In this case, the studied networks had values of the scaling exponent around 2, and an average clustering coefficients (see section 3) about an order of magnitude larger than expected for scale free networks. This scaling exponent around 2, suggests that these networks are probably under a dynamical regime between that of an ordered system and the one of a chaotic one. This regime is known as *critical* and it has been observed in many different complex systems (Shmulevich et al., 2005). Another important theoretical contribution of this work is the introduction of the *topological overlap matrix* (Ravasz et al., 2002; Cheng et al., 2019).

The **interactome** (Sanchez et al., 1999) is a useful concept related to Protein-protein (physical) interaction (PPI) networks, which are also organized into functional subnetworks or modules. An interactome is defined as a biological network, which encompasses the complete set of molecular interactions in a particular cell. These interactions range from physical (as in PPI networks) to indirect, as is the case of epistatic or gene-gene interactions, and may even include edges defined by regulatory interactions like those of a GRN (Gómez-Romero et al., 2020). Even if interactomes seem to be less clearly defined than other biological networks, they may be used to represent processes that, although not completely understood, may be associated with some specific phenotypes. The *human disease network* (HUDiNE) (Goh et al., 2007) was actually created by using interactomes. HuDiNe, according to its creators is *a network of disorders and disease genes linked by known disorder–gene associations*. The observation that genes linked to similar diseases present a higher likelihood of sharing physical interactions between their products (e.g., PPI) and a higher correlation in their expression profiles, lead to the conclusion that such a network will likely display characteristic disorder-specific functional modules. This fact was corroborated by analyzing the topological structure of the HuDiNe (Goh et al., 2007). Since the release of HUDiNE, interactomes related to disease have been carefully curated and archived in structured databases, thus making possible the discovery of new *co-morbidities* from a molecular rather than epidemiological perspective (Menche et al., 2015).

In the case of human diseases, modular network decomposition has been applied to further our understanding of the interactions driving the emergence of several complex diseases (Sardiu et al., 2017; Tripathi et al., 2019; Lucchetta and Pellegrini, 2020). One good example is the work of De Matos Simoes and collaborators with cancer cells. By using a network modularity analysis, they showed that transmembrane proteins along with ion channel complexes and receptors play a significant role in the pathogenesis of B-cell lymphoma. The authors based their argument on the observation that central and peripheral layers in the modular decomposition of the networks may play different physiological roles. Hierarchical modular separation may then provide clues as to cross-regulatory phenomena in complex phenotypes. Specifically, they noted that these molecules act via the communication disruption between the intracellular regions and the peripheral regions of B cells (de Matos Simoes et al., 2012). In pancreatic cancer, the disruption of intracellular adhesion and cell-division cycles in the tumors were found to be driven by clearly defined transcriptional modules (Long et al., 2016). Also, network communities related to survival have been found in regulatory networks from hepatocellular carcinoma (Xu et al., 2016). Expression activity of the genes in such modules may contribute to timely stratification and tumor staging of liver cancer patients.

Other complex phenotypes have been dissected by analyzing the community structure of their underlying networks. During brain development, for example, it has been shown that the perinatal transition leads to modular reorganization of the brain, which is in turn associated with the development of new functions. This modularization is also correlated with specific gene sets whose expression are synchronously changing, as they share transcriptional regulators (Monzón-Sandoval et al., 2016). Similar methods have allowed the identification of distinctive molecular pathways that differentiate early and late-onset temporal lobe epilepsy in children (Moreira-Filho et al., 2015). These studies have pointed out that differentially expressed modules in early onset epilepsy are related to neural excitability and febrile seizures, whereas no neural excitability gene modules were found for late onset. These findings support the hypothesis that early onset epilepsies, even if accompanied by severe hippocampal damage, may present compensatory effects. This difference may set the basis for differentiated drug treatments.

Community structure in regulatory networks may also be useful to discover potential molecular targets to treat complex diseases (Muraro and Simmons, 2016). In coronary artery disease, for instance, modules associated with the hypertrophic cardiomyopathy pathway and membrane-related functions were detected (Liu et al., 2016). These pathways, the authors suggest, can provide a means to define a set of druggable process-specific targets (Ashrafian et al., 2011). Transcriptional modules associated with the response to allergens leading to seasonal allergic rhinitis have been also identified by Shi and collaborators (Shi et al., 2010). These modules revealed that the MAP kinase, B-cell receptor and toll-like receptor signaling pathways are crucial for the critical stages of allergic rhinitis. Regarding the role of gene regulation on viral pathogenicity and how it has been shaped by modular adaptation, it has been discussed how enhanced redundancy leads to robustness of the infectious phenotypes (Oliveira et al., 2013).

So far we have discussed several examples where finding modules in biological networks lead to a better understanding of the molecular and regulatory processes involved in certain phenotypes and behaviors. A relevant fraction of the modularity finding approaches used in network biology were developed with a particular biological question in mind. The methods thus developed were, in general, efficient to answer that kind of questions but resulted somehow lacking generalizability. We call these methods *ad hoc*, since they have been developed for a special purpose. Most of these methods are indeed quite useful on a case-by-case basis. However, since modularity analysis is a relevant problem in contemporary theoretical biology, it is desirable to have general methods, or at least methods with broad applicability, to help lay the conceptual foundations of biological modularity. We believe that a first step toward this aim consists in applying the general methods developed in graph theory and network science to biological questions and fine-tune them to account for known biological phenomena. In the next section, we will review several necessary concepts and useful methods for modularity detection that come from a more theoretical perspective. As such, these methods were developed to be useful under any, or at least several, quite general circumstances. We have also included a benchmark section, where we discuss how these algorithms stand against each other in the discovery of modules using both real and synthetic datasets. Although the field of modularity detection in biological systems is somewhat young, it has a long history in physics, and thus, many algorithms are already out there making impossible to review all of them. A later section will discuss the most relevant methods separated by the algorithm they are based on in the hopes that the reader will find some of them useful for their research.

## 3. Network Theory

In order to better understand the modularity detection methods that will follow, we will briefly define/recall a few important network properties. For a deeper coverage of these and several other properties we suggest the reader to look, for instance, at the review by Newman (2010). For an introductory lecture on the importance of networks in biology and their main applications besides modularity detection we suggest the review by Green et al. (2018).

### 3.1. Complex Networks: Concepts and Definitions

For the sake of clarity, we will briefly introduce some well-known definitions of network theoretical concepts.

**definition 1.***A **network** is formally defined as a graph *G*(*V, E*) over two sets: a set of nodes or vertices, *v*_*i*_ ∈ *V*, (e.g., bio-reactants), and a set of edges or links connecting such vertices (*e*_*i*_ ∈ *E*) (e.g., chemical reactions). The connectivity of the network is often represented by the **adjacency matrix*** 𝔸 = **A*_*i,j*_, where _*i,j*_ ≠ 0 implies an existing interaction between nodes *v*_*i*_ and *v*_*j*_*.

**definition 2.***The degree-distribution of a network refers to the distribution of the number of connections per node, and is defined as the number of connections a given node has to other nodes (called the *degree* of the node). Thus, **the degree distribution** is defined as the probability distribution of the degrees of all the nodes of the network. This measure is often used as an indicator of the relative importance of a particular node (Barabasi and Oltvai, 2004)*.

*Mathematically: Let*$v_{i}^{m}$*be the set of vertices connected to a given vertex (a.k.a. node) *m* (i.e.,*$A_{i,m}\neq 0;\forall v_{i}\in v_{i}^{m}$*). We call*$v_{i}^{m}$ the **neighborhood** of vertex *m*. *The size, or cardinality, of this set* $C\left(e_{i}^{m}\right)=k_{m}$ *is called the **degree** or **connectivity** of vertex *m*, also written as *deg*(*v*_*m*_)*.

**definition 3.***A **Network motif** is defined by a group of connected nodes (a sub-graph) that is prevalent in a network or in several networks. Each motif is thus associated with a particular pattern of interconectedness between vertices, and may reflect a framework in which particular functions are achieved efficiently. These patterns describe arrangements of interconnection that are present with a significantly higher frequency than in networks where nodes are randomly connected (Milo et al., 2002)*.

**definition 4.***Intuitively, **network modularity** consists in associating network nodes to different categories or subsets of the network. Assignment is based on connectivity patterns within the graph, rather than on some inherent node features. The formal definition of network modularity is still controversial, but we believe that by giving some enclosing definitions from graph theory, we can gain a deeper understanding of this concept and methods described below*.

**definition 5.*****Full/Overlapping partition**. We may consider a set *Z* of disjoint subsets of a network *Z*(*V, E*) so that*$Z=Z_{1}\dot{\cup }Z_{2}\dot{\cup }\ldots \dot{\cup }Z_{k}$. *This is called a *full partition* of the network. If, on the other hand, we allow a non-empty intersection between the subsetsZ*_*i*_ ⋂ *Z*_*j*_ ≠ ∅, *we have* $Z=\hat{Z_{1}}⋃\hat{Z_{2}}⋃\ldots ⋃\hat{Z_{k}}$ *which is called an *overlapping partition* of the network*.

**definition 6.*****Incomplete/Modular Partition**. We can also consider an incomplete partition of *Z*, i.e., one in which not every vertex in *V* is assigned to a subset. In this case we callM* ⊂ *Z* *a modular partition of the network*, *M* = *M*_1_ ⋃ *M*_2_ ⋃ …⋃*M*_*k*_ ⊂ *Z*. *The subsets *M*_*i*_ (which may or may not be overlapping) are called the *modules* of *Z*. There are several ways in which a network can be partitioned. Here lies the difficulty in defining modularity in complex networks: different definitions of modularity may induce different modular partitions of the network, which leads to different modularity measures*.

**definition 7.***The **clustering coefficient** *CC*(*i*) for a particular vertex *i* in a network is given by*:


(1) $$\begin{array}{l} CC\left(i\right)=\frac{number\;of\;triangles\;connected\;to\;i}{number\;of\;possible\;triangles\;connected\;to\;i} \end{array}$$

*Here, a triangle is a set of three fully interconnected nodes. Since 0 ≤ *CC*(*i*) ≤ 1. Equation (1) can be rewritten as*:


(2) $$\begin{array}{l} CC\left(i\right)=\frac{2E_{i}}{k_{i}\left(k_{i}-1\right)} \end{array}$$

*Where *E*_*i*_ is the number of triangles centered in vertex *i* and *k*_*i*_ is the degree of that vertex*.

*Once we have an operative definition of clustering coefficient, its mean value is the average over all nodes i*.


(3) $$\begin{array}{l} \langle CC\rangle =\frac{1}{N}\overset{N}{\underset{i}{\sum }}CC\left(i\right) \end{array}$$

〈*CC*〉 *is a probabilistic measure of the abundance of triangles (not necessarily triads, but also higher order motifs) in the network*.

Global measures such as the 〈*CC*〉 are computationally cheap (Fortunato, 2010). However, their utility is mostly restricted to the case of hierarchic modularity scenarios (modules within modules). Hierarchic modularity was originally defined as the property of self similarity in the module distribution in a large scale network, evidenced by a power-law behavior of the clustering coefficient *C*(*k*) ~ *k*^−1^. This relation in turns involves the coexistence of a hierarchy of nodes with different degrees of *node-modularity* –as measured by the node-specific clustering coefficient–. In brief, under such assumptions, the higher a node connectivity *k* is, the smaller its clustering coefficient, which in the asymptotic regime gives rise to the inverse law, 1/*k*.

### 3.2. Network Models: Types and Approaches

#### 3.2.1. Weighted Networks

A weighted network is defined by the assignment of a weight for each of the edges of the network. These weights are established based on the type and strength of the interaction at hand. Interestingly, weighted networks have proven to further increase the reliability of the modules proposed. For instance, the weighted overlap measure (WOM) is a similarity measure that calculates the overlap between two sets weighted by their relative contribution to the overall (joint set) (Smith, 1985). The WOM has been used to define gene modules that are more cohesive than those obtained through unweighted networks though this is not always the case. Here a more *cohesive* module means that the average value of the inter-module clustering coefficient is higher than the average value of the network's clustering coefficient. Since its proposal, the WOM has been used to recover experimentally validated functional gene modules in cancer cells and in yeast (Zhang and Horvath, 2005). More importantly, it has been shown that modularity affects biological functions as the dynamics of the whole network is determined by the organizational patterns generated by the modules themselves. For example, bi-stable switches, where weighted edges are essential for bi-stability, are known to enhance regulatory feedback and feed-forward loops, which in turn are related to the ability of an organism to adapt to changing environments (Kashtan et al., 2009; Gyorgy and Del Vecchio, 2014).

The functional role of regulatory modules has proved to go beyond that of loops and motifs. By studying a transcriptional network of myeloid cells, Alcalá-Corona and coworkers showed that modules are consistently associated at the pathway level to sets of biological functions (Alcalá-Corona et al., 2016). Community structure has also proven to affect the dynamical behavior of the network (Qi and Ge, 2006). By analyzing simple models of gene regulation, Xu and Wang were able to fully decompose a complex network in terms of independent functional modules (Xu and Wang, 2010). Although clear cut decompositions are not likely to occur in a real biological networks due to pleiotropy, decompositions make possible to observe modular effects in an idealized way. For instance, they have been used to study the effects of the free scale topology and of hierarchical modularity on the large scale structure of GRNs (Zhan, 2007). When network structural properties are supplemented with appropriate dynamic behavior, robustness is enhanced (Aldana et al., 2007). This increase in robustness has been shown to be due to the presence of large attractor basins that lead to stable gene expression patterns (Sevim and Rikvold, 2008).

#### 3.2.2. Multi-Level Networks

The advancement of graph theory along with interactomes gave rise to the concept of multi-layered networks. Multi-layered networks encompass several types of interactions and node types. However, in this *multiplex framework* interactions are integrated into different network layers and therefore more information about the real underlying phenomena can be retained (Didier et al., 2015). Adding extra dimensions to a graph can make the associated mathematical analyses more intricate and hinder the application of common topological approaches to study modularity. Nevertheless, it has been shown that real modules encountered in curated networks are better recovered with modular algorithms applied to multilayered networks, compared with the same algorithms applied to single-layer networks. A detailed mathematical framework for multilayer networks—introductory, though not elementary—is found in the comprehensive paper by De Domenico et al. (2013).

In addition to the multiple molecular levels of description of a phenomenon, multi-layered networks can be adapted to include multiple species which can be useful in disciplines such as in comparative genomics. This extended approach also has more robust scalability features than mono-layered networks (Ritchie et al., 2016). Multi-layered networks have enforced the development of new theoretical approaches need for discovering modularity such as the *Multiplex PageRank algorithm* (Iacovacci and Bianconi, 2016).

Another important feature of multi-layered networks is that they allow a direct analysis of the functional features of their subjacent modules (e.g., pathway-based strategies). This approach is useful for studying phenotypes that are naturally multi-layered, like those associated with genetic regulation where multiple different sources (e.g., transcription factors, chromatin, methylation, etc.) are responsible for the phenotype. For instance, through the use of a multi-layered network of transcription factors and microRNA co-targeting, along with protein-protein interaction and gene co-expression (Cantini et al., 2015) were able to find a set of cancer driver genes associated with the community structure of the network.

A related issue to that of multilayer networks is *multiscale modularity*. Despite highly connected nodes, or hubs, are often labeled as the most important nodes of a network, recent studies in the modular structure of the regulatory networks of *Escherichia coli, Saccharomyces cerevisiae*, and *Staphylococcus aureus* revealed an unexpected relevance for low degree metabolites. By using flux balance analysis and graph theoretical methods, Samal et al. (2006) were able to discover connected clusters of low-degree metabolites. These large clusters of low degree nodes turned out to be over-represented in these metabolic networks so that a majority of the essential metabolic reactions could be characterized by just a few low degree metabolites. In this study, reactions whose fluxes were strongly correlated formed well-defined communities in metabolic networks of the organism. The large scale community structure, that is, the network modules conforming relatively large subnetworks, and the small scale modularity (partitions of small motifs), represent a complex interplay that has been shown to play an important role in metabolism under the assumption of hierarchical network organization (Gao et al., 2016). By introducing the concept of multiscale modularity, they propose that network community structure may be defined in several organizational levels, taking into account high and low degree nodes.

## 4. Modularity Detection Algorithms

From the perspective of the statistical physics, computer science, computational sociology, network science and complex systems communities, there has been a significant amount of work devoted to solve the modular partition or community detection problem. Unlike what happened with biological networks, these methods aimed at reaching formal and theoretically-founded results with wide applicability. It is important to note that there is the possible drawback of losing some interpretability of the results in the quest for generality. However, it is our belief that these methods will prove useful for the biological community, as these approaches remain largely unknown and offer complementary views of the same problem. With this in mind, the following sections will be focused on introducing this second perspective to the community detection problem.

Classification of community detection algorithms depends on their approach to the graph partition problem. Although there is a wide variety of methods and algorithms to approach the problem of graph partitioning and network modularity detection, they often fall in one of five (quite general and sometimes overlapping) possible categories:

1. Methods based on data clustering
2. Methods based on optimization of the modular partition
3. Methods based on the spectral properties of the adjacency matrix
4. Random walk based and other dynamical algorithm methods
5. Stochastic block models

As we will see, there are advantages, disadvantages and limitations in all types of models. For this reason, it is wise to consider the features, applicability and benchmark performance before opting-in for a certain model.

### 4.1. Data Clustering-Based Methods

There are several methods based on measuring some significant statistical similarity or distance over the biological data. Some techniques have been developed to ascertain whether a set of proposed modules adequately represents the whole set of molecular determinants of a single disease, or closely related diseases.

For instance, in Menche et al. (2015), a topological method was devised in order to locate disease-related communities within the interactome (whole set of interactions in a particular cell). This method uses the overlap among communities of different pathologies to predict disease-disease associations. Although simple, this method has proved very useful and further improvements have been made to the initial algorithm, in particular on relation to the establishment of endo-phenotype models as discussed in Ghiassian et al. (2015) and Ghiassian et al. (2016).

One important limitation of clustering based methods rely on the challenge to determine the optimal number of clusters. The problem of an optimal number of clusters/modules is actually an open challenge in theoretical computer science and graph theory. Even approximate solutions often depend on the specifics of the algorithm used. Some methods as the ones based on spectral bisection have conditions to define an a priori number of clusters, while other methods like those based on structural properties, on dynamical process over the networks and those which have a stochastic component; may determine a number of clusters, based on their large and local structure of the network, an approach some consider to be more *natural*.

One relevant method for disease module detection is DIAMOND (Ghiassian et al., 2015). The theoretical ground for DIAMOND is that in incomplete interactomes “*diseases cannot be associated with topologically dense network communities”*, rather, the statistical significance of an interaction, meaning the weight of the link, is the relevant quantity used to characterize such modules. This highlights the impact of the node/link ratio in the establishment of interacting structure and then in biological function. By extending the ideas of the DIAMOND/HuDiNe approaches it is possible to analyze the relationship between drug targets and disease-proteins through a topological *proximity measure*. This measure quantifies the interactions between drugs and disease-proteins in the human disease interactome (Guney et al., 2016) and can be used as a proxy for therapeutic effect. This can be useful for establishing a basis for drug screening and repositioning and evaluation strategies. Another approach to detect modularity in the interactome was based on identifying joint patterns of gene expression and drug response (Chen and Zhang, 2016). This was done to gain further insight into the biochemical mechanisms of drug action that may drive the development of new therapeutic targets in cancer. Interactome modularity has allowed *de novo* design of therapeutic strategies in cancer and also allowed the creation of methods for drug repositioning analysis (Chen et al., 2016). Such methods are aimed at detecting multi-targeted drug candidates that may disable malignant cellular functions.

Several methods have been proposed to analyze community structure in PPI networks. Feature selection by clustering has been applied to real and synthetic interaction data revealing modules with increased biological significance for *E. coli* and yeast networks (Henriques and Madeira, 2016). A similar approach was used in the NCMine method (Tadaka and Kinoshita, 2016) which is implemented as a plug-in for the popular network visualization and analysis suite Cytoscape (Adamcsek et al., 2006; Su et al., 2010; van Dongen and Abreu-Goodger, 2012) and is based on a technique called near-clique mining that distinguishes nodes in a network as either “core” or “peripheral” to a given subnetwork. Topological Data analysis (TDA) has also been used to detect topological network modules in protein interaction networks. TDA encompasses several statistical methods like clustering and perturbation analysis to find structure in data. By deleting protein complexes of the *S. cerevisiae* INO80 protein interaction network and performing TDA, isolated modules that contain proteins with shared biological functions were discovered to belong to the same module, even if they mapped to distinct locations of the network (Sardiu et al., 2017).

Clustering using genetic algorithms has been also applied with certain success (Ramadan et al., 2016). In brief, an objective function is built for exclusive clustering (nodes belonging to a unique module) and overlapping clustering (a particular node or set of nodes can be as indicated by spectral clustering methods, see section 4.2). This function is then optimized by a replication/mutation/recombination genetic algorithm in order to detect modular components of the network identified as protein complexes. One approach to detect such modularity in GRNs is through phylogenetic profiling. This approach is based on the idea that the joint presence or joint absence of two traits across various species is used to infer a meaningful biological connection, such as involvement of two different proteins in the same biological pathway.

As it was mentioned, sometimes approaches made use of hybrid methods, such is the case, for instance, of the work by Servis and Clark (2021) that perform a cluster identification strategy by using modularity optimization to analyze chemical heterogeneity in complex solutions. We will abound on modularity optimization in the next subsection.

### 4.2. Methods Based on *Modularity* Optimization

Unlike the methods based on similarity of data, most of the methods take into account the large-scale structure of the network itself, defined by the edges between nodes, regardless of the source of the data (Newman, 2012). Such as the case of the methods based on and supported by some class of Modularity optimization (see Definition 8).

In order to categorize different modularity measures, we must distinguish between *local* and *global* methods that quantify and assess network modularity. Measures of local modularity emphasize scoring specific clusters or partitions of the network. This score considers the number of modules that are dense or sparsely connected in a given assignment (Reichardt and Bornholdt, 2006). The more dense connections are within a module and the more sparse the connections are from within a module to outside vertices, the higher the modularity score will be. The local modularity of a network is usually given as the score of the highest-scoring partition. Finding the best partition and evaluating its score solves the modularity problem completely, but it relies on comprehensive enumeration of partitions, a problem that often carries computationally prohibitive combinatorial burdens (Fortunato, 2010).

The case of *global* modularity of a network is different in the sense that global measures usually are computed without *a priori* computing the network partitions. Instead, this measure relies on other network properties such as the *average clustering coefficient* 〈*CC*〉. The rationale is that vertices that form a module should have adjacent neighbors, as they increase the modular density and induce the formation of *triangles* in the graph.

An important family of local modularity measures is based on the concept of *edge-betweenness*, a concept introduced to generalize the node-associated betweenness centrality measure. Edge betweenness is then defined as the number of shortest paths between pairs of nodes that run along a given edge. The more paths traverse pairs of nodes traversed by an edge, the more *central* the edge is for the global connectivity structure of the network (Freeman, 1977). The first algorithm that used this concept was proposed by Girvan and Newman (Newman and Girvan, 2004) and is a paradigmatic example of the application of local modularity measures. The method consists in disconnecting sets of vertices by removing edges with larger betweenness. This algorithm was applied to several simulated networks as well as a number of real networks with an *a priori* known modular structure with good overall performance. More importantly, Newman and Girvan also provided a formal measure of network modularity.

**definition 8.***Given a network modular partition we have the following*:


(4) $$\begin{array}{l} Q=\underset{i}{\sum }\left(e_{ii}-a_{i}^{2}\right)=Tr\left(𝔼\right)-||{𝔼}^{2}|| \end{array}$$

*Here, *e*_*ij*_ is the matrix element –from the modularity matrix* 𝔼– *whose entries are defined as the fraction of all the edges in the network that connects nodes in the *i* module to the nodes in the module j*, $a_{i}=\underset{j}{\sum }e_{ij}$. *Notice that, for an arbitrary matrix* 𝕏, *a norm is defined as* $||𝕏||=\underset{i}{\sum }\underset{j}{\sum }x_{ij}$.

*Q* is called the *Girvan-Newman modularity* of a network partition, or sometimes just the *Modularity*. *Q* measures the fraction of edges in the network connecting vertices within the same module or *community* (or *intra-community edge* ratio) and then subtracts form this fraction its expected value in a network with the same partition scheme over randomly connected nodes. *Q* = 0 implies that the partition's modularity is not better than random, whereas *Q* = 1 is indicative of a strong modular structure.

Modularity can also be rewritten (Clauset et al., 2004) as:


(5) $$\begin{array}{l} Q=\frac{1}{2m}\underset{i,j}{\sum }\left[A_{ij}-\frac{k_{i}k_{j}}{2m}\right]\delta \left(C_{i},C_{j}\right) \end{array}$$

Where *m* is the total number of edges in the network. *k*_*i*_ is the degree for node *i*. *A*_*ij*_ is the adjacency matrix. *C* is an indicator function such that *C*_*i*_ = *C*_*j*_ implies that nodes *i* and *j* belong to the same community, δ is Kronecker's delta function. This way, if two nodes *i* and*j* belong to the same community δ(*C*_*i*_, *C*_*j*_) = 1, otherwise δ(*C*_*i*_, *C*_*j*_) = 0.

There is yet another (equivalent) way to represent the modularity *Q* that may result even more useful in practice (Fortunato and Barthelemy, 2007; Porter et al., 2009):


(6) $$\begin{array}{l} Q=\overset{M}{\underset{s=1}{\sum }}\left[\frac{l_{s}}{L}-{\left(\frac{d_{s}}{2L}\right)}^{2}\right] \end{array}$$

The sum, over all *M* modules of the partition, *l*_*s*_ is the number of edges inside community *s*. *L* is the number of edges in the network and *d*_*s*_ is the total degree of nodes in module *s*.

These important ideas lead to the establishment of *Community Detection* as one of the foundational problems of Network Science (Newman and Girvan, 2003; Newman, 2004a; Kovács and Barabási, 2015). Maximization of modularity *Q* has been proposed as a central idea in several optimal network partition algorithms (Clauset et al., 2004; Newman, 2004b, 2006b). However, modularity optimization, also known as *Q*_*max*_ algorithms, are constrained by a resolution limit that depends on the overall size of the network and on the interconnection density of the modules, which may lead to failure of *Q*_*max*_ methods due to sub-optimal optimization caused by the presence of a multitude of local minima on the modularity function (Fortunato and Barthelemy, 2007).

A related issue with respect to large networks is that calculating the modularity score *Q* (see Equation 6) belongs to the family of NP-Hard or non-deterministic polynomial-time problems. The main characteristic of these problems is that they cannot be solved in polynomial-time, so they are computationally and time consuming, precluding its direct use on extremely large networks. Several heuristic approaches have been proposed to deal with this problem (Danon et al., 2005; Duch and Arenas, 2005; Guimera and Amaral, 2005; Newman, 2006b; Von Luxburg, 2007; Brandes et al., 2008). One particularly useful technique is known as the Louvain method (Blondel et al., 2008). This approach is based on a two-step heuristic: (1) a maximal modularity full partition is obtained by merging nodes in order to maximize modularity through a greedy method, (2) then a network is formed in which nodes are the modules from the first step. This stage is continued recursively until no further improvement in modularity can be obtained.

A whole new family of methods was developed after the introduction of the modularity measure *Q*. Most of these methods aimed to maximize either *Q* itself or some proper function of *Q* under the rationale that if one is able to find a partition that maximizes *Q*, the induced community structure would be optimal. In this family we can find the original works by Newman (2004b) as well as later refinements of his method, either by himself (Clauset et al., 2004; Newman, 2006b) or by others (Guimera et al., 2004; Duch and Arenas, 2005; Blondel et al., 2008; De Leo et al., 2013). However, since maximization of the *Q*-measure has a resolution limit that depends on the size of the network and the degree of interconnection between the modules, the method is not fail-safe (Fortunato and Barthelemy, 2007; Lancichinetti and Fortunato, 2011). Some recent implementations, however, have been developed to improve the results obtained under *Q*-optimization as is the case of the works by Medus and Dorso (2009), Khadivi et al. (2011), Gong et al. (2011), and (Bettinelli et al., 2012).

### 4.3. Spectral Graph Theory

Another family of algorithms is based on *Spectral graph theory*, which uses the analysis of the eigenvalues of the *adjacency matrix* or the *Laplacian matrix* of a graph. It consists in a transformation of the set of nodes into a set of points in a space whose coordinates are elements of eigenvectors, then the set of points can be clustered via standard techniques (Fortunato, 2010). The change of representation induced by the eigenvectors makes the cluster properties much more evident (Donath and Hoffman, 1972; Fiedler, 1973).

**Figure 1:**
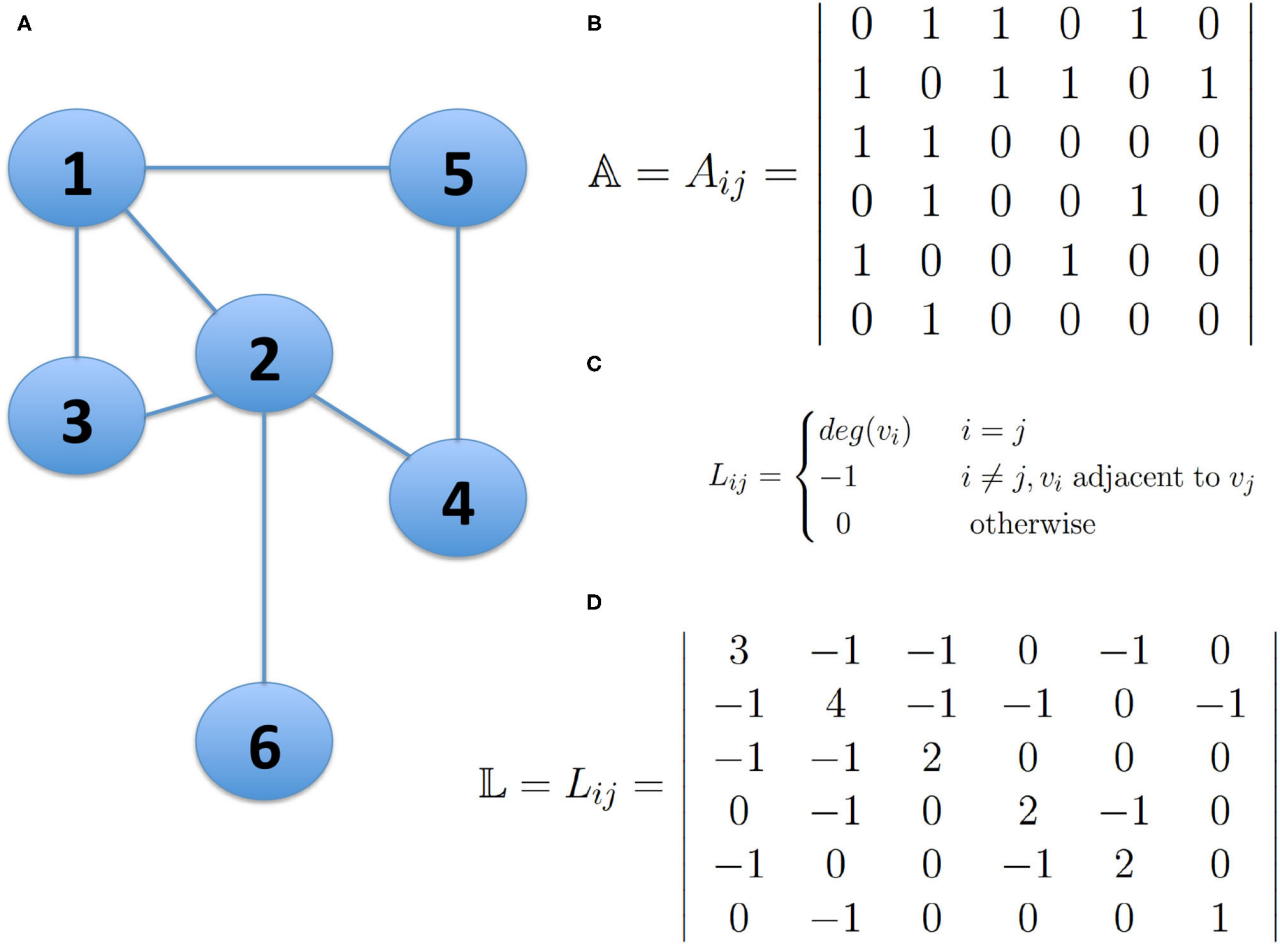
The Laplacian Matrix of a network. Panel **(A)** presents a small undirected network; Panel **(B)** shows the Adjacency Matrix 𝔸 describing the network connectivity of the network in **(A)**; Panel **(C)** shows the definition of the Laplacian Matrix of a Network and panel **(D)** shows the Laplacian Matrix 𝕃 of the network in **(A)**. The bold numbers represent the degree of node i, whenever i=j. This figure is intended for illustrative purposes, no *actual results* are presented.

The analysis of the spectrum of the **Laplacian matrix** 𝕃, is the most used approach in spectral clustering. This matrix can be derived from the adjacency matrix 𝔸 of a network and it is constructed by reversing the signs of the non-diagonal entries and replacing the diagonal entries with the degree of the corresponding node (See Figure 1).

The Laplacian matrix can be written in block-diagonal form, that is, the nodes can be ordered in such a way that the Laplacian displays *k* square blocks along the diagonal, with some entries different from zero, and all other elements vanish. Each block is the Laplacian of the corresponding subgraph, so it has the trivial eigenvector $\vec{1}$ with components (1, 1, 1, …, 1, 1). Therefore, there are *k* degenerate eigenvectors with equal non-vanishing components in correspondence with the nodes of a block, whereas all other components are zero. In this way, from the components of the eigenvectors, it is possible to identify the connected components of the graph, and then based on this property, it is possible to find highly connected groups of nodes and the expected number of modules in which the network may be partitioned.

Since the values of the eigenvector components are close for nodes in the same community, it is possible to use them as coordinates, such that vertices turn into points in a metric space. So, for *M* eigenvectors, the nodes can embed in an *M*-dimensional space. Thus, modules appear as groups of points well-separated from each other (Donetti and Muñoz, 2004). Also, it is possible to use the Laplacian matrix property, in which, if the graph has *g* connected components, the largest *g* eigenvalues are equal to 1, with eigenvectors characterized by having equal-valued components for nodes belonging to the same component. Thus, the modules can be found by inspecting the components of the eigenvectors with eigenvalue 1 (Capocci et al., 2005).

Furthermore, in the context of *Spectral clustering*, there is a remarkable relationship introduced by Newman (Newman, 2006b), between *Modularity optimization* and the spectral properties of the *adjacency matrix* known as *Spectral optimization*. We can rewrite the *Q* optimization in terms of finding the spectrum of a particular matrix as we will see below.

Starting from Equation (5), it is possible to define the *modularity matrix* *B*_*ij*_ as:


$$\begin{array}{l} 𝔹=B_{ij}=\left(A_{ij}-\frac{k_{i}k_{j}}{2m}\right) \end{array}$$

Now, let us suppose a particular *a partition* of a network into just **two** modules. Thus we can assign to each node, a quantity *s*_*i*_, such as:


$$s_{i}=\{\begin{array}{ll} +1, & \text{if a node }i\text{ belongs to group 1}\\ -1, & \text{if vertex }i\text{ belongs to group }2 \end{array}$$

Thus, *Q* can conveniently be written in matrix form:


(7) $$\begin{array}{l} Q=\frac{1}{4m}\underset{ij}{\sum }B_{ij}s_{i}s_{j}=\frac{1}{4m}{\vec{s}}^{T}𝔹\vec{s} \end{array}$$

where $\vec{s}$ is a column vector whose elements are *s*_*i*_.

Then, in order to optimize this form of *Q* it is possible to perform the so-called *relaxation method* (that is, allowing its entries to take continuous values and retaining the norm of the vector), which is one of the standard methods for the approximate solution of vector optimization problems such as this one. Thus, by differentiating and imposing the constraint $|s|=\sqrt{n}$ or equivalently:


$$\begin{array}{l} \underset{i}{\sum }s_{i}^{2}=n \end{array}$$

The modularity maximization problem is now straightforward. We now have a maximization problem with this norm as a constraint, or equivalently, $\left(n-\underset{i}{\sum }s_{i}^{2}\right)=0$. This is done by introducing a *Lagrange multiplier* λ, and taking the partial derivative with respect to the components of the vector (one at a time) of the following expression:


(8) $$\begin{array}{l} \frac{\partial }{\partial s_{i=k,j=k}}\left[\underset{i}{\sum }\underset{j}{\sum }B_{ij}s_{i}s_{j}+\lambda \left(n-\underset{i}{\sum }s_{i}^{2}\right)\right]=0 \end{array}$$

to obtain:


(9) $$\begin{array}{l} \left[\underset{i}{\sum }B_{ik}s_{i}+\underset{j}{\sum }B_{kj}s_{j}-2\lambda s_{k}\right]=0 \end{array}$$

which leads to:


$$\begin{array}{l} \underset{j}{\sum }B_{kj}s_{j}-\lambda s_{k}=0 \end{array}$$

$$\begin{array}{l} \underset{j}{\sum }B_{kj}s_{j}=\lambda s_{k} \end{array}$$

for all *k*.

Which is in a matrix form an eigenvalue problem for the *modularity matrix*:


(10) $$\begin{array}{l} 𝔹\vec{s}=\lambda \vec{s} \end{array}$$

The value of λ that maximizes *Q* is the largest possible one, that is the dominant eigenvalue of the matrix 𝔹.

It is worth mentioning, that similarly to this approach, the **spectral bisection method** (Barnes, 1982), uses the spectrum of the Laplacian matrix, to find partitions of a graph by dividing it recursively into two groups. Every partition of a graph with *n* nodes in two groups can be represented by an index vector $\vec{s}$, whose component *s*_*i*_ is +1 if a node *i* is in one group and *a*1 if it is in the other group. Then the cut size *R* of the partition of the graph in the two groups can be written as:


(11) $$\begin{array}{l} R=\frac{1}{4}{\vec{s}}^{T}𝕃\vec{s} \end{array}$$

Finally, the *Modularity optimization* approach can be extended to a more than two modules, by writing an additional contribution Δ*Q* to the modularity upon further dividing a group *g* of size *n*_*g*_ in two as:


(12) $$\begin{array}{l} \Delta Q=\frac{1}{4m}\underset{i,j\in g}{\sum }\left[B_{ij}-\delta_{ij}\underset{k\in g}{\sum }B_{ik}\right]s_{i}s_{j} \end{array}$$

(13) $$\begin{array}{l} \Delta Q=\frac{1}{4m}{\vec{s}}^{T}{𝔹}^{\left(g\right)}\vec{s} \end{array}$$

where δ_*ij*_ is Kronecker's δ, and 𝔹^(*g*)^ is the *n*_*g*_ × *n*_*g*_ matrix with elements indexed by the labels *i, j* of nodes within group *g*. Because Equation (13) has the same form as Equation (7) it is possible to apply the spectral approach to this generalized *modularity matrix*, just as before, to maximize Δ*Q*.

In addition, the *modularity matrix* 𝔹 also has always the trivial eigenvector $\vec{1}$ with eigenvalue zero (like the *Laplacian matrix*), because the sum of the elements of each row/column of the matrix vanishes. Thus, it is also possible to optimize modularity on bipartitions via *spectral bisection*, by replacing the Laplacian matrix with the modularity matrix (Newman, 2006a,b).

### 4.4. Random Walk Based Models

The use of random walks to find modules on a network is based on the somehow intuitive premise that a random walker moving on the network will spent more time inside modules—due to the high density of edges, thus many possible trajectories—than hoping from one module to another. A first approach to this problem was addressed by Zhou (2003) who used random walks to define a *distance* between pairs of nodes, assuming that there is a high likelihood that *closer* nodes—under this measure of distance—belong to the same module. Such distance was used to define global and local *attractor nodes* used to detect modules, i.e., minimal distance subnetworks. A different but related approach was taken by Pons and Latapy (2006) on a method called *Walktrap*. Here, distance is calculated via the probability that a random walk moves from one module to another on a fixed number of steps, then grouping nodes via hierarchical clustering.

A method based on the application of the Markov property of node-to-node walks called Markov Cluster algorithm (MCL) was developed by Van Dongen (2001). MCL simulates a diffusive process in the network. A *stochastic matrix* is obtained by dividing every entry of the adjacency matrix *A*_*ij*_ by the corresponding degree of node *i*. This stochastic matrix is used to calculate transition probabilities on a Markov random field. This method is quite elegant and comparatively easy to implement, however, its large computational complexity makes it difficult to apply in practice for real (large) networks (even in sparse cases).

As already mentioned, for large sparse networks also the standard versions of spectral based algorithms are suboptimal, in the sense that in some cases these fail to detect communities even when other algorithms such as belief propagation can do so. Efforts to improve these spectral theory methods have been made by resorting again to random walk dynamics, mainly through implementing non-backtracking random walks (the random walker cannot move backwards) over the network (Krzakala et al., 2013; Newman, 2013; Zhang and Newman, 2015). Other methods in the literature are built on ideas borrowed from non-linear dynamic processes, such as spin-coupling models with nearest neighbor interaction (Reichardt and Bornholdt, 2004), synchronized oscillators (Arenas et al., 2006; Arenas and Diaz-Guilera, 2007), as well as generalized random walks (Van Dongen, 2001; Zhou and Lipowsky, 2004; Pons and Latapy, 2005). Among this plethora of models, INFOMAP has been shown to be quite reliable and computationally efficient (Rosvall and Bergstrom, 2007, 2008).

The INFOMAP algorithm is founded on a clever combination of random walk dynamics and information theory. The main idea is to reach optimal compression of the information needed to describe the diffusion process of a set of random walkers. This is achieved by using the random walk *itself* as a proxy for the diffusion process via a sequential enumeration algorithm and the use of tools of information theory and computational linguistics.

In a nutshell, the approach is quite similar to the way we imprint location information on geographic maps of cities: you can map a large number of close-to-each-other streets into a neighborhood (“a module,” with its own description) and a series of close-by neighborhoods into a town. The larger the scale of these *urban modules*, the smaller the total amount of information needed for their description. In a similar way, the INFOMAP algorithm looks up for the *minimal description length* for the modular partition of a network. The best partition is the one that can be described with the minimal information.

In brief, the *description length* is a measure of the complexity of a given process. By using the description length is possible to characterize the trajectory of a random walk (or the trajectories for an ensemble of random walkers), in the form of the *map equation*:


(14) $$\begin{array}{l} L\left(M\right)=q_{↶}H\left(Q\right)+\overset{m}{\underset{i=1}{\sum }}q_{↶}H\left(P_{i}\right) \end{array}$$

Here, *L*(*M*) is the description length of an ensemble of random walkers moving through a given modular partition *M*. The first term $q_{↶}H\left(Q\right)$ represents the average number of bits needed to describe the movements from nodes in one module of the partition to nodes in another module, whereas the second term represents the information for the intramodule walks. Since by the coding theorem (Knuth, 1985), the information needed to characterize inside module walks is smaller, a minimal description length implies that most of the time walkers move inside modules of a given partition, thus optimizing modularity, allowing however for the presence of a number of intermodule hops. This method uses a *greedy* algorithm, so it can be applied quite efficiently even to large networks, directed or undirected. There are also INFOMAP implementations to find hierarchic modular structure (Rosvall and Bergstrom, 2011) and overlapped modules (Esquivel and Rosvall, 2011).

### 4.5. Stochastic Block Models

Statistical inference provides a powerful set of methodological tools useful in modularity detection. The usual way to proceed is by adjusting a *generative network model* to the experimental data. A stochastic block model (SBM) is by far, the most used model to generate networks with a modular structure. The essentials of the SBM are as follows:

The stochastic block model generates a number *n* of vertices of the network; the algorithm makes a partition of the vertex set {1, …, *n*}{1, …, *n*} into *q* disjoint subsets *C*_1_, …, *C*_*q*_ i.e., the modules. By starting with a symmetric *q* × *q* matrix *P* containing edge probabilities for all the possible connections. These probabilities must be known a priori. Then the SBM is generated by randomly sampling this edge set as follows: any two vertices *u* ∈ *C*_*i*_ and *v* ∈ *C*_*j*_ are connected by an edge with probability *P*_*ij*_.

Modularity detection works out by optimizing the unnormalized log-likelihood that a given partition *g* of a graph *G* in *q* modules will be reproduced by the SBM (Karrer and Newman, 2011).


(15) $$\begin{array}{l} L\left(G|g\right)=\overset{q}{\underset{i,j=1}{\sum }}e_{ij}\log\left(\frac{e_{ij}}{n_{i}n_{j}}\right) \end{array}$$

Here L(G|g) is the log-likelihood for a partition *g* of a given network *G* to be produced by the standard SBM. *e*_*ij*_ is the number of edges connecting module *i* with module *j* of the partition, and *n*_*i*_, *n*_*j*_ are the number of nodes in modules *i* and *j* respectively. The sum includes the case *i* = *j*. The strongest drawback of the method is that it requires *a priori* knowledge of the number *q* of modules in which the network has to be partitioned, although this limitation has been recently overcome by using a Bayesian formulation (Peixoto, 2018).

General SBM models (i.e., non-Bayesian) have been demonstrated to be formally equivalent to modularity optimization approaches that do not usually require a fixed number of modules for the partition (Newman, 2013). Despite this and the fact that maximum likelihood exact estimation is an *NP* problem—so all solutions are approximate—SBM models are still popular in statistics and machine learning algorithms.

As we have discussed in this section, topology based methods for modularity detection are robust, general and intelligible. They can also be benchmarked with experimentally available modular partitions. Such validation uses robust statistics, such as the ones given by normalized mutual information measures. The strength of these methods is that they do not rely *a priori* on any non-topological information, as they are based on the (weighted or un-weighted, directed or un-directed) connectivity as given by adjacency matrices. This is the basis of their generality and broad applicability, in particular to complex biological problems.

The fact that these methods do not need any prior knowledge—aside from the connectivity structure—does not preclude us to incorporate such information when available, to enhance our intuition and empower our predictions when applied to real large scale biological networks. For this reason we strongly believe that the popularization of these approaches within the computational and systems biology research settings will prove to be highly beneficial for both, the construction of more general approaches to study modularity in biology and for the further development of analytic methodologies in the theory of complex networks.

## 5. Benchmarking and Performance Tests

Whenever several methods perform a similar task, benchmarking becomes necessary. However, as described in Tripathi et al. (2016), a large heterogeneity among different community structure discovery methods is often found. As many of the available methods for module discovery have been developed as *ad-hoc* solutions, they often lack reliability when applied to other biological systems. Also, the intrinsic complexity of biological modularity makes it hard for a single method to describe all types of modules correctly. Nevertheless, in the following section we will show how by resorting to theoretically sound and rigorous methods of comparison that do not rely on the specifics of a given biological system, one can attain precise measurements of performance for any module detection method.

### 5.1. Testing Performance and Scoring Measurements

Benchmarking community detection algorithms using real biological networks is not optimal, as it is not clear what the ideal partition is. However, real networks such as the social network of bottle-nose dolphins from Doubtful Sound (New Zealand) built and studied by Lusseau (2007), as well as the network of college football teams obtained by Girvan and Newman (Girvan and Newman, 2002) have been used for this purpose. Real biological network communities (also called *ground-truth communities*) are often inferred from non-topological studies carried out by network curators, which based on experimental observations (e.g., protein-protein interactions) define the network itself. As these methods rely only on observed data, it is possible that the resulting network is either incomplete or has spurious interactions. So how can one find these modules and relate them to particular functionalities, especially when such functionalities are unknown? One general approach is to use random network methods to test if the community or modular structure in our networks is valid and significant (Sah et al., 2014). One common approach consists in generating network models that satisfy the constraints imposed by the real networks (such as the connectivity, the number of nodes, etc.) and keep a graph structure that is as random as possible. These network realizations allow the use of a large set of tools already available to analyze the topology of random networks. In particular, they are useful for creating *null-models* that serve as a baseline to which we can compare the significance of our partition model. As such null models have been established, they can be used to test biological functional hypotheses. This generation of null models serves directly to generate scoring metrics that allow the comparison and selection of the best network partitions. These null-model networks may be generated synthetically, and this way we could test to what extent the algorithm is able to found the a-priori known communities.

There are two classic and widely used performance tests for community detection algorithms: the GN and the LFR (Fortunato, 2010), both of which belong to a class of methods generated under the *planted* *l-partition model* (Condon and Karp, 2001).

**definition 9.***In the **planted l-partition model** a network with *n* = *g* · *l* nodes, is partitioned into *l* groups of *g* nodes each. Nodes in the same group are linked with a fixed probability *p*_*in*_, whereas nodes in different groups are linked with probability *p*_*out*_. Each module is then a random Erdös-Rényi network with *p* = *p*_*in*_ and if every module were a node, the whole network would also be an Erdös-Rényi graph with *p* = *p*_*out*_*.

*For a subgraph representing a module or community *C*, the average connectivity degree will be given as 〈*k*〉_*in*_ = *p*_*in*_(*g*−1) and the average external degree would be 〈*k*〉_*out*_ = *g* · *p*_*out*_(*l*−1) (recall that for an Erdös-Rényi graph connected with probability *p*, the average degree is given as 〈*k*〉 = *p*(*n*−1)). If these conditions hold, the average degree for the whole network is*


(16) $$\begin{array}{l} \langle k\rangle =p_{in}\left(g-1\right)+g\cdot p_{out}\left(l-1\right) \end{array}$$

*This way, if 〈*k*〉_*in*_ > 〈*k*〉_*out*_ (i.e., if the intra-module average degree is greater than the inter-module average degree), then the network will have well-defined community structure. This is equivalent to the intuitive definition of modularity, namely *p*_*in*_ > *p*_*out*_*.

The GN test was designed by Girvan and Newman (Girvan and Newman, 2002) to test their community detection algorithm. It is a particular case of the *planted* *l-partition model* where the authors fixed *l* = 4 and *g* = 32 to get a network composed of 128 nodes forming 4 modules with 32 nodes each and an average degree of 〈*k*〉 = 16. Within this framework link-density is adjusted by scanning the values of the average in-degree 〈*k*〉_*in*_ and out-degree 〈*k*〉_*out*_ to choose specific values to change the community structure for each network provided that 〈*k*〉 = 〈*k*〉_*in*_ + 〈*k*〉_*out*_ = 16.

Under this model it is possible to have explicit expressions for the average in- and out- degrees, namely: 〈*k*〉_*in*_ = *p*_*in*_(*g*−1) = 31*p*_*in*_ and 〈*k*〉_*out*_ = *g* · *p*_*out*_(*l*−1) = 96*p*_*out*_. By varying the values of *p*_*in*_ and *p*_*out*_ it is then possible to simulate networks with a stronger or weaker modularity. For instance, a clearly defined community structure is induced if *p*_*in*_ ≃ 0.5 or larger, whereas a value of *p*_*in*_ ≃ 0.25 or lesser precludes the existence of well-defined modules.

For this benchmark communities are well-defined for 〈*k*〉_*in*_ > 8. One of the advantages of the GN test is that by varying a single parameter in a pretty simple network it is possible to contrast different network partition methods. In order to test a particular method via the GN test one has to calculate a *similarity measure* between the partition of the GN network as given by this method against the natural partition of the network in four modules of the same size. A highly used similarity measure—proposed by Newman and Girvan (Girvan and Newman, 2002)—is the fraction of edges correctly classified, though a more objective measure can be the normalized mutual information between partitions (see Equation 17) (Arenas et al., 2008).

In spite of its simplicity and mathematical rigor, the GN test presents a couple of important shortcomings derived from unrealistic assumptions. First, all the nodes are expected to have the same degree. Second, all the communities must be of the same size. Clearly real complex networks, such as those encountered in biology, are characterized by long-tailed degree distributions or power law-like ones, and also by heterogeneous community sizes. Some improved versions of the GN method have been developed such as the one presented in Fan et al. (2007) where different weights are assigned to *inner* and *outer* edges, regarding their position in the communities.

The fact that the planted *l*-partition model generates mutually-interconnected Erdös-Renyi random graphs implies that all the nodes will have almost the same degree and all the communities will have exactly the same size. Of course, these two features do not match with what is observed in real networks. To tackle this problem, Lancichinetti et al. proposed the *LFR Benchmark test* (Lancichinetti et al., 2008). The LFR test assumes that the node degree distribution and the module size distribution follow a—more realistic—power law behavior. Each node shares a fraction 1 − μ of its edges with nodes within its community and a fraction μ with nodes in other communities. Hence 0 ≤ μ ≤ 1 the mixing parameter is equivalent to a normalized version of the 〈*k*〉_*out*_ used in the GN test. The LFR test was devised for undirected, unweighted networks, but there are implementations for directed, weighted graphs including the possibility to have overlapping communities (Lancichinetti and Fortunato, 2009a). Aside from purely computational costs, the main performance test for network community detection algorithms must establish a clear criterion to compare the degree of *similarity* between the modules discovered (i.e., the specific partition) by an algorithm and the real (in the test, a priori known) partition. There are several proposals in the complex network literature as how to measure similarity between different partitions (Meilă, 2007), some of them based on pair recounting and group coincidence counts (Fortunato, 2010).

Additionally, two widely used measures are the fraction of correctly classified edges and the normalized mutual information between partitions. The former was proposed by Girvan and Newman to test their algorithm, but can be generalized to other benchmark tests. The criteria for the correct classification is as follows: Each of the modules *A*_*i*_ of the partition found by the given algorithm is compared to all of the *actual* modules *B*_*i*_, known a priori from the real network partition. When more than half of the nodes in one of these *A*_*i*_ correspond to those of a community *B*_*i*_ then *A*_*i*_ is considered to be correctly classified and no more comparisons between *A*_*i*_ and the rest of the *B*_*i*_s are carried out. In the contrary case (less than half corresponding nodes) or when the community *A*_*i*_ is smaller than half the size of the given *B*_*i*_, then the module is compared to the rest of the *B*_*i*_'s until exhaustion. This criterion is quite stringent since there are cases in which one may consider that some of the nodes have been correctly classified by the algorithm but the measure (total node count divided by the size of the network to give a number between 0 and 1) rules them out.

**definition 10.***The **normalized mutual information between partitions** (NMIBP) was proposed by Danon et al. as a similarity measure (Danon et al., 2005) built on ideas proposed by Ana and Jain (2003), Kuncheva and Hadjitodorov (2004)*.

*The rationale is that if two partitions are similar, very little information is needed to infer one partition given the other. One is able to calculate the mutual information between two partitions *A* and *B* by building a confusion matrix ℕ where rows correspond to the *actual* modules and columns correspond to the modules found by the given algorithm. The *N*_*ij*_-th element of ℕ is the number of nodes in a real (known a priori) community *i* that are also present in the community *j* detected by the algorithm. Since the partitions under comparison may have a different number of groups (the modules or communities), ℕ is not necessarily a square matrix. This way the similarity between two partitions *A* and *B* is given by the normalized mutual information measure (NMI) as follows*:


(17) $$\begin{array}{l} NMI\left(A,B\right)=\frac{-2\sum_{i=1}^{C_{A}}\sum_{j=1}^{C_{B}}N_{ij}log\left(\frac{N_{ij}N}{N_{i.}N_{.j}}\right)}{\sum_{i=1}^{C_{A}}N_{i.}log\left(\frac{N_{i.}}{N}\right)+\sum_{j=1}^{C_{B}}N_{.j}log\left(\frac{N_{.j}}{N}\right)} \end{array}$$

*Here, the number of actual modules (partition *A*) is denoted by *C*_*A*_, the number of modules found by the algorithm (partition *B*) is *C*_*B*_, the sum over the row *i* of the matrix* ℕ = *N_*ij*_ is *N*_*i*._ and the sum over column *j* is *N*_.*j*_ and *N* is the total number of nodes. If the partitions *A* and *B* are identical, then NMI*(*A, B*) = 1, *whereas completely dissimilar partitions give NMI*(*A, B*) = 0.

*This measure is highly used in the performance tests for community detection algorithms since it is highly sensitive as it quantifies explicitly the amount of information recovered by the algorithm from the original topological structure of the network (Lancichinetti and Fortunato, 2009b; Lancichinetti et al., 2011; Tripathi et al., 2016). The NMIBP measure can be used in the GN and LFR performance tests, both in standard and overlapping partitions (Lancichinetti and Fortunato, 2009a)*.

More recently there have been some other approaches that propose new benchmarks that provide actual techniques to determine which is the most suited algorithm in most circumstances based on observable properties of the network under consideration. Also considering the use of the mixing parameter μ and the Normalized Mutual Information measure (NMI) (Yang et al., 2016). There are also benchmarks based on novel methods that generate networks with topological properties found in empirical biological networks (Sah et al., 2014; Gilbert, 2015).

Despite the high performance of algorithms and methods shown on the artificial networks generated by benchmarks and its test with the μ (mixing factor), for example on the LFR test, an open question is, whether the methods with good results on benchmarks necessarily find meaningful modules in actual networks (Jebabli et al., 2018; Cherifi et al., 2019).

It may happen that the community structure found by some methods with high performance in benchmarks, does not necessarily correspond to correct ground-truth community structure—that is, the one based on real known node groups, or derived from some metadata or even identified by the node attributes—and vice versa. There could be a substantial difference between structural communities and metadata groups (Orman et al., 2012; Hric et al., 2014; Jebabli et al., 2018).

So, for a fair assessment of the performance of some methods, it is necessary to have a good match between the detected partition and the attribute-based partitioning for considering that a method is reliable. Both tests are complementary, and we recommend applying both of them to perform a complete and accurate assessment of an actual community structure.

Nonetheless, to overcome these limitations, exploiting the topological features of the so-called “*community graphs*” (where the nodes are the communities and the links represent their interactions) has been proposed to evaluate the algorithms; in contrast with metrics defined at node level that are fairly insensitive to the variation of the overall community structure. Thus, if the ground-truth community structure is available, it is possible to compare it vs. the one discovered by these algorithms by using these clustering-based metrics as has been proposed by some authors (Orman et al., 2012; Hric et al., 2014; Jebabli et al., 2018; Cherifi et al., 2019), where more emphasis has been put on the topology of the community structure.

In this direction, some modifications to the LFR benchmarks have been proposed to make generated networks more realistic (Orman et al., 2012). In this work, authors studied generated networks in terms of community-centered topological properties to evaluate some methods, they used such properties to compare community structures to rank the tested community detection algorithms. As well, recently da Fonseca Vieira et al. (2020) tested some representative state-of-the-art methods for overlapping community detection (Cherifi et al., 2019) with synthetic and real-world benchmark *Ground-Truth networks* showing that, although the methods can identify modular communities, they often miss many structural properties of the communities.

### 5.2. Good Performance Methods Commonly Applied to Biological Networks

Beyond presenting the benchmarking for the performance of the different algorithms, it is important to point out which methods we think are good for finding modules, given the biological question under consideration. The question of which algorithm is the best for biological networks is not easy to answer, it will depend on the context of the research question and the data on which the network is built.

However, two of these graph-theoretically-grounded, general purpose algorithms have been widely applied in biological networks with good and significant results, such methods are the **Louvain** (Blondel et al., 2008) and **Infomap** (Rosvall and Bergstrom, 2008). Both methods have good performance and accuracy scores, as we can see from the several artificial network bencharmking analyses (Lancichinetti et al., 2008, 2009; Lancichinetti and Fortunato, 2009a; Sah et al., 2014; Gilbert, 2015; Yang et al., 2016), as well as in *Ground-Truth networks* and also in terms of *community-centered topological properties* (Orman et al., 2012; Hric et al., 2014; Jebabli et al., 2018). In addition, both methods show good results and performance in biological networks, even in comparison with more recent methods (Mall et al., 2017b; Debnath et al., 2021). Furthermore, they also have been proved as standard methods to identify biologically meaningful modules in biological networks (Zheng et al., 2021) and even for evaluating significant topological differences between networks (Mall et al., 2017a). In addition, they have been incorporated on different Bioinformatic analysis suites and tools, as well as implemented in different programming languages widely used today, such as R, Python, MatLab, and C++ and incorporated into standard widely network analysis libraries such as igraph.

The *Louvain method* (Blondel et al., 2008) is by far the most widely used method in biological networks, showing significant results and meaningful modules (Praneenararat et al., 2011) even compared with newer methods in recent studies (Şen et al., 2014; Bennett et al., 2015; Rahiminejad et al., 2019; Calderer and Kuijjer, 2021). The method is indeed still widely used nowadays, for example, in the context of SARS-COV-2 analyses (Zheng et al., 2020). The efficiency and high performance of this method lie on its taking into account the whole structure of the network and searching for the best partition in an algorithmic greedy fashion. In addition, this method has been extended and applied to bipartite biological networks (Pesantez-Cabrera and Kalyanaraman, 2016; Calderer and Kuijjer, 2021) as well as to multilayer and multiplex biological networks (Mucha et al., 2010; Didier et al., 2015; Mittal and Bhatia, 2018).

On the other hand, *Infomap* is accepted as a very well-known method in module detection (Acharya et al., 2012) and even as a method for comparing the performance and accuracy of novel methods in biological networks (Lecca and Re, 2015), and has been incorporated in some bioinformatic layouts as a standard community detection framework (Aldecoa and Marín, 2014; Zhou and Xia, 2018; Farage et al., 2021). Moreover, has been widely adapted and extended by its authors in several ways to different kinds of networks and problems in community detection, for example, hierarchical module detection (Rosvall and Bergstrom, 2011), bipartite networks (Kheirkhahzadeh et al., 2016) and multilayer networks (De Domenico et al., 2015). In addition, these extensions have proved to give meaningful results in the context of biological networks as ecological networks (Pilosof et al., 2020; Farage et al., 2021), multiplex genetic datasets (Mittal and Bhatia, 2018) and breast cancer networks (Alcalá-Corona et al., 2018a). The efficiency and high performance of Infomap lie in how information flow in a network can reveal the structure of it (Esquivel and Rosvall, 2011; Aslak et al., 2018; Eriksson et al., 2021), combined with a strategy of optimizing partitions such as the *Louvain method*, which make it one of the most robust and applicable methods for all kinds of networks and giving meaningful results (Kawamoto and Rosvall, 2015; Emmons and Mucha, 2019).

Finally, it is worth mentioning that other three methods have been demonstrated to be efficient and reliable in the context of biological networks in comparison with Infomap and Louvain: the **Spinglass Method** (Reichardt and Bornholdt, 2004, 2006), **OSLOM** (Lancichinetti et al., 2011), and **Label Propagation approach** (Garza and Schaeffer, 2019).

Thus, we can suggest **as a general strategy for community detection in biological networks to apply both Louvain and Infomap, in addition to one of these three latter methods and then consensing the partition by the Consensus Clustering approach** (Lancichinetti and Fortunato, 2012) to compute a unique community structure.

## 6. Application Example: Community Detection Methods for Cancer Networks

Network approaches have been extensively used for instance, to observe structural differences between cancer and non-cancer related networks (Reyna et al., 2020; Wang et al., 2020). These differences, often carry functional features that may help to understand such complex phenotypes (Miecznikowski et al., 2016; Drago-García et al., 2017; de Anda-Jáuregui et al., 2019; Dorantes-Gilardi et al., 2020).

Finding functional modules in cancer has been a matter of intense research. A common method to infer such modules resorts to the so-called *Weighted gene co-expression network analysis (WGCNA)* (Zhang and Horvath, 2005; Langfelder et al., 2008). In this method, Pearson correlation is used to evaluate pairwise gene co-expression. Such co-expression network can be decomposed into modules by using different methods.

For instance, in Ai et al. (2020), the authors used the dynamic tree cut method (Langfelder and Horvath, 2008) to infer modules in a microarray-based colorectal cancer (CRC) gene co-expression network. This method improves the classic hierarchical clustering that sets a fixed cutoff value. A dynamic branch cutting depending on the dendrogram shape is implemented. With this approach, Ai and cols., found that GUCA2A, GUCA2B, and CDH3 genes were highly correlated with the occurrence of CRC.

Along similar lines, WGCNA was used to analyze 182 CRC and 54 normal samples (Qiu et al., 2020). There, a k-means clustering was used to find modules, and the hub genes from those modules were separated into samples with high and low expression. The authors identified that overexpression of MYL9, MYLK, and CNN1 genes was associated with poorer outcome in CRC patients.

In breast cancer, efforts have been made to observe modules that may be underlying functional processes (Wilkinson and Huberman, 2004; Zhu et al., 2008; Cantini et al., 2015). It is widely known that breast cancer is a highly heterogeneous disease. This heterogeneity can be traced down to the genetic level (Alcalá-Corona et al., 2017).

Molecular subtyping provides a helpful tool to classify tumors by identifying common patterns in their genetic expression. One of the most used classification methods is PAM50 (Sørlie et al., 2001). Samples are grouped based on the molecular signature. With this method, breast cancer can be divided into four main differentiated subtypes: Luminal A, Luminal B, HER2+, and Basal-like. Each subtype has a different clinical and histopathological manifestation.

Network approaches to identify modules in breast cancer molecular subtypes has been a matter of intense research. For instance, the infomap algorithm has been used to reveal functional modules in HER2+ breast cancer transcriptional network (Alcalá-Corona et al., 2018b). Additionally, it has been observed that in the HER2+ tumors related network, a hierarchical modular structure appears (Alcalá-Corona et al., 2018a).

In basal-like breast cancer, network modularity has been used to observe functional modules and discern whether or not those modules are shared between the cancer and the non-cancer network (de Anda-Jáuregui et al., 2019). It has been observed that the basal breast cancer has a different distribution of module size between cancer and non-cancer networks (de Anda-Jáuregui et al., 2019). Additionally, those modules are composed of different genes.

In all those cases, cancer networks are formed by small connected same-chromosome gene components. Often, said components coincide with modules independent of the community detection method. However, this is not always the case. For example, in García-Cortés et al. (2021), for Luminal A breast cancer, an RNA-Seq-derived gene co-expression network was decomposed into communities by using four different methods: Fast greedy (Clauset et al., 2004), Infomap (Rosvall and Bergstrom, 2008), Leading eigenvector (Newman, 2006b) and Louvain (Blondel et al., 2008).

The aforementioned methods have different postulates and different approaches to detect communities. In that work (García-Cortés et al., 2021) it was demonstrated that, independent of the algorithm used to detect communities, the results were very similar in terms of the number of detected communities and the nature of the genes observed in each community.

Despite modules being quite similar, independently of the method to detect them (Jaccard indexes between modules obtained by the different methods, are larger than 0.95), the algorithm with optimal modularity was the Louvain method. Interestingly, Modularity is larger in the case of Luminal A network than the healthy network, for all methods.

An additional effect observed when comparing cancer and non-cancer derived networks, is a high proportion of same-chromosome gene-gene interactions in cancer phenotypes. On the other hand, healthy tissue-derived networks are composed of interactions between genes from any chromosome in a homogeneous fashion. This phenomenon has been called *loss of long-distance co-expression in cancer* (Espinal-Enríquez et al., 2017). This abrupt change has been reported for different tissues such as breast cancer (Espinal-Enríquez et al., 2017; de Anda-Jáuregui et al., 2019), each breast cancer molecular subtype (García-Cortés et al., 2020), clear cell renal carcinoma (Zamora-Fuentes et al., 2020), lung adenocarcinoma and lung sqamous cell carcinoma (Andonegui-Elguera et al., 2021). It is worth noticing that modularity has been used as an indirect measure of coordinated gene function (Solé et al., 2002; Segal et al., 2003; Lee et al., 2004; Tanay et al., 2004; Zhu et al., 2008). In this case, modules do not always represent gene function, but often act as a proxy for *spatial clustering* between genes from the same chromosome.

The studies just mentioned are just a handful instances, illustrating how network modularity determination is a becoming an essential approach to biological discovery.

## 7. Concluding Remarks

As we have already discussed, complexity in biological systems can be understood partially by using network approaches. Modularity is often an inherent component of complex biological networks. However relevant, network modularity discovery (or community detection, as is also called) is a daunting task. Its importance in theoretical biology, to describe the emergence of functional behaviors in biological systems, as well as its use in understanding the underlying principles behind such functionality make it a worthy tool in biology.

In the past years, a number of relevant approaches to this problem have been developed in the computational and systems biology settings. Most of these approaches, although extremely informative are built upon *Ad Hoc* assumptions and are thus not easy to generalize. Hence, they provide useful information, but are too specific. On then other hand, the network science and statistical physics research communities have been developing a series of quite general modularity detection algorithms. Here we present some of them, organized as *families* of methods, depending on their methodological foundations: (i) clustering algorithms, (ii) modularity optimization methods, (iii) methods based on the spectral properties of adjacency matrices, (iv) methods based on random walks and (v) methods based on stochastic block models. These broad families of methods along with the benchmarks that have been developed to evaluate their performance may constitute a relevant toolbox for the analysis of biological systems from a more general perspective. We argue that by resorting to these methods (freed from the design constraints typical of *Ad Hoc* methods) will allow to focus on the actual biology rather than on the method's specificities.

The problem of modularity and the discovery of functional communities in biological networks is an important emerging field of research. Omic high throughput technologies and the rise of computing power as well as the development of novel analytical algorithms have allowed the generation of bio-molecular network models at an unprecedented pace. This has led us with the need to develop theoretical and computational tools to extract biologically useful (e.g., functional or mechanistic) information from such large scale models. A wide variety of biological questions that can be answered—at least partially—by knowing the modular structure of the underlying networks, are being added to the current research scenario in the systems biology and genomics communities. A number of powerful mathematical and computational schemes to deal with modularity are also currently under development.

In the preceding review, we have discussed both, the biological problems and the computational approaches to the problem of modularity in complex bio-molecular networks. It is our sincere desire that works like this will stimulate the discussion between researchers in all the involved fields. A discussion that may in turn strengthen the ties of collaboration and ultimately leads to fruitful cross-fertilized scientific discoveries.

## Author Contributions

SA-C and EH-L: conceived the idea, contributed to the writing of the manuscript, and revised the manuscript. SS-M and JE-E: contributed to the writing of the manuscript and revised the manuscript. All authors contributed to the article and approved the submitted version.

## Funding

This work was supported by CONACYT (grant no. 179431/2012), as well as by federal funding from the National Institute of Genomic Medicine (Mexico). Additional support has been granted by the National Laboratory of Complexity Sciences (grant no. 232647/2014 CONACYT). SS-M appreciates the support from CONACyT through the Catedras-CONACyT program. EH-L also acknowledges additional support from the 2016 Marcos Moshinsky Research Chair in the Physical Sciences. The funders have no role in the design or development of this work.

## Conflict of Interest

The authors declare that the research was conducted in the absence of any commercial or financial relationships that could be construed as a potential conflict of interest.

## Publisher's Note

All claims expressed in this article are solely those of the authors and do not necessarily represent those of their affiliated organizations, or those of the publisher, the editors and the reviewers. Any product that may be evaluated in this article, or claim that may be made by its manufacturer, is not guaranteed or endorsed by the publisher.
